# Plasma insulin-like growth factor binding protein-3 proteolysis is increased in primary breast cancer

**DOI:** 10.1054/bjoc.2001.1860

**Published:** 2001-07

**Authors:** S I Helle, S Geisler, T Aas, T Paulsen, J M P Holly, P E Lønning

**Affiliations:** Department of Oncology and; Surgery, Haukeland University Hospital, Bergen, N-5021, Norway; Division of Surgery, Bristol Royal Infirmary, Bristol, BS2 8HW, UK

**Keywords:** insulin-like growth factor binding protein-3, proteolysis, insulin-like growth factor-I, primary breast cancer

## Abstract

Fasting blood samples were obtained before definitive surgery or biopsy in 128 patients referred to the department of surgery with suspected or manifest breast cancer. Insulin-like growth factor (IGF)-I, IGF-II and free IGF-I were measured by radioimmunoassay/immunoradiometric assay, while IGFBP-3 proteolysis was evaluated by Western immunoblot. 12 patients had ductal carcinoma in situ benign conditions, while staging revealed metastatic disease in 15 of 16 patients with invasive cancers. IGFBP-3 proteolysis above the normal range was recorded in 19 patients with invasive cancers, but in none of the patients suffering from DCIS/benign conditions. Increased IGFBP-3 proteolysis was most frequently recorded in patients harbouring large tumours and metastatic disease (Stage I: 0/19, 0%; Stage II: 3/45, 7%, Stage III: 9/37, 24%, and Stage IV: 7/15, 47%). IGFBP-3 proteolysis was significantly higher in Stage III (*P* =0.01) and IV (*P*< 0.001) patients compared to the other stage groups (*P* = 0.001). IGF-I and IGF-II correlated negatively to IGFBP-3 proteolysis and age. Plasma levels of IGF-I and -II were significantly lower in patients with elevated IGFBP-3 proteolysis compared to those within the normal range. Our findings reveal alterations in the IGF-system among a substantial number of patients with large primary breast cancers. © 2001 Cancer Research Campaign http://www.bjcancer.com


*British Journal of Cancer* (2001) **85**(1), 74–77
© 2001 Cancer Research Campaign
doi: 10.1054/ bjoc.2001.1860, available online at http://www.idealibrary.com on IDEAL®

http://www.bjcancer.com

### Plasma insulin-like growth factor binding protein-3 proteolysis is increased in primary breast cancer

**SI Helle[1], S Geisler[1], T Aas[2], T Paulsen[2], JMP Holly[3] and PE Lønning[1]**

[1]Department of Oncology and [2]Surgery, Haukeland University Hospital, N-5021 Bergen, Norway; [3]Division of Surgery, Bristol Royal Infirmary, Bristol BS2 8HW, UK

**Summary** Fasting blood samples were obtained before definitive surgery or biopsy in 128 patients referred to the department of surgery with suspected or manifest breast cancer. Insulin-like growth factor (IGF)-I, IGF-II and free IGF-I were measured by radioimmuno-assay/immunoradiometric assay, while IGFBP-3 proteolysis was evaluated by Western immunoblot. 12 patients had ductal carcinoma in situ benign conditions, while staging revealed metastatic disease in 15 of 16 patients with invasive cancers. IGFBP-3 proteolysis above the normal range was recorded in 19 patients with invasive cancers, but in none of the patients suffering from DCIS/benign conditions. Increased IGFBP-3 proteolysis was most frequently recorded in patients harbouring large tumours and metastatic disease (Stage I: 0/19, 0%; Stage II: 3/45, 7%, Stage III: 9/37, 24%, and Stage IV: 7/15, 47%). IGFBP-3 proteolysis was significantly higher in Stage III ($P = 0.01$) and IV ($P < 0.001$) patients compared to the other stage groups ($P = 0.001$). IGF-I and IGF-II correlated negatively to IGFBP-3 proteolysis and age. Plasma levels of IGF-I and -II were significantly lower in patients with elevated IGFBP-3 proteolysis compared to those within the normal range. Our findings reveal alterations in the IGF-system among a substantial number of patients with large primary breast cancers. © 2001 Cancer Research Campaign http://www.bjcancer.com

**Keywords**: insulin-like growth factor binding protein-3; proteolysis; insulin-like growth factor-I; primary breast cancer

Insulin-like growth factors (IGF)-I and -II are potent mitogens to breast cancer cell lines and may prevent apoptosis (Karey and Sirbasku, 1988; Resnicoff et al, 1995). The type I IGF-receptor (IGF-IR), which mediates these signals, is found to be expressed in most human breast cancers (Peyrat et al, 1988), and its expression is enhanced by oestrogens in ER+ breast cancer cell lines (Wiseman et al, 1993; Huynh et al, 1996).

Several studies have suggested a role for the IGFs in breast cancer growth and tumour development. High plasma levels of total IGF-I have been associated with an elevated risk for breast cancer in premenopausal women (Hankinson et al, 1998) and also for prostate cancer in males (Chan et al, 1998). Other investigators have reported alterations in the IGF-system in breast cancer patients including elevated levels of total IGF-I, reduced levels of IGF-binding protein-3 and an elevated IGF-I/IGFBP-3 ratio compared to healthy individuals (Peyrat et al, 1993; Bruning et al, 1995). Recently, increased expression of the type I IGF receptor in the tumour has been related to local relapse following radiotherapy in breast cancer patients (Turner et al, 1997).

Most plasma IGF-I and -II are bound in a ternary complex consisting of IGFBP-3 and an acid-labile subunit (ALS). Due to its long half-life (Guler et al, 1989), this complex is thought to act as a depot of IGF-I and -II in plasma (Jones and Clemmons, 1995). Proteases having IGFBP-3 as substrate may increase the bioavailable fraction of IGF-I by decreasing the binding affinity of IGFBP-3 for the growth factors (Blat et al, 1994), resulting in a drop in plasma total IGF-I levels (Cotterill et al, 1996).

*Received 27 November 2000*
*Revised 5 March 2001*
*Accepted 20 March 2001*

*Correspondence to:* PE Lønning

An increased IGFBP-3 protease activity has been described in certain pathological conditions like diabetes (Bereket et al, 1995), severe illness (Davies et al, 1991), but also in patients suffering from advanced malignancies, including breast cancer (Frost et al, 1996). If this also occurs for patients with early breast cancer, it could make total IGF-I an unreliable predictor of bioavailable IGF-I in these patients. Previous findings from our group have also indicated IGFBP-3 proteolysis to correlate to tumour burden in metastatic breast cancer (Helle et al, 1996). An elevated protease activity might have a detrimental positive feedback effect on tumour growth by facilitating the release of IGF-I to the tissues (Blat et al, 1994).

The main purpose of this study was:

(1) To determine whether plasma IGFBP-3 proteolysis was elevated in patients with primary breast cancer.
(2) If so, to test the hypothesis that an elevated IGFBP-3 proteolysis might correlate to tumour burden.
(3) To evaluate the possible influence of IGFBP-3 proteolysis on plasma levels of free IGF-I.

### PATIENTS AND METHODS

### Patients

A total of 128 women referred to the breast unit at the Department of Surgery, Haukeland University Hospital due to suspected or manifest breast cancer were enrolled in the study.

Age, stage distribution, and treatment of the patients are shown in Table 1. As blood samples were obtained before definitive histological diagnosis and staging were performed, 12 patients turned out to have a benign condition or ductal carcinoma in situ while 15 patients had metastatic disease at primary diagnosis. None of the stage IV patients had any history of breast cancer

**Table 1** Age and stage distribution of the patients included in the study. *n* = number of patients in each group. Age is given as its median value (with range). DCIS; ductal carcinoma in situ

| Stage (AJCC/ UICC) | *n* | Age (years, median with range) | Subgroups | Treatment |
|---|---|---|---|---|
| Benign | 8 | 46.5 (35–59) | | Excision |
| Stage 0 (DCIS) | 4 | 67.5 (46–76) | | Excision |
| Stage I | 19 | 69 (46–86) | | Lumpectomy[a] / mastectomy |
| Stage II | 45 | 66 (39–88) | IIA (*n* = 19) | Lumpectomy[a]/mastectomy |
| ($T_2N_1$)[2] | | | IIB (*n* = 26) | Lumpectomy/mastectomy + adjuvant therapy |
| | | | | Primary chemotherapy + surgery (T3N0)[a b] |
| Stage III | 37 | 64.5 (55–73) | IIA (*n* = 21) | Primary chemotherapy + surgery[a b] |
| | | | IIB (*n* = 16) | Primary chemotherapy + surgery[a b] |
| Stage IV | 15 | 63 (55–73) | | Chemotherapy or endocrine treatment depending on oestrogen receptor (ER) status, age, and disease severity |

[a]Patients treated with lumpectomy, patients with more than 4 lymph nodes with tumour infiltration, patients with T3 tumour, and all stage III patients received postoperative radiation therapy. [b]Patients with ER positive tumours received adjuvant treatment with tamoxifen, age < 55 years received chemotherapy.

before referral. Noteworthy, the stage distribution in our study does not reflect the stage distribution of breast cancer in the Norwegian population in general, as the Department of Surgery is a referral unit having patients with locally advanced disease in particular referred from other clinics. While none of the patients had any uncontrolled metabolic or other serious disease at the time of study inclusion, 7 patients suffered from different diseases that could possibly have an influence on the IGF-system. These included rheumatoid arthritis (2 patients), ulcerative colitis (1 patient) or type II diabetes (4 patients).

Each patient gave her informed consent before inclusion. The protocol was approved by the Regional Ethical Committee.

### Blood sampling

All patients had fasting blood samples obtained between 07.30 and 08.30 am at the day of biopsy or definitive operation. Patients with locally advanced disease (T3–4) or $T_2N_2$ had samples collected before start of chemotherapy. Samples were collected in heparinized vials. Centrifugation was performed within 30 minutes and plasma stored at –20°C until analysis.

### Control group

As a control group we prospectively collected plasma samples from 114 normal postmenopausal women attending a mammography screening program. Women with a history of diabetes or endocrine disease were excluded. Median age was 66 years (range 55–72). We also obtained fasting plasma samples from 39 healthy premenopausal women from the hospital staff with median age 31 years (range 23–43). The normal ranges of the IGF-parameters are based on the 95% confidence intervals of the individual observations.

### Assays

Plasma levels of IGF-I and IGF-II were measured by RIA following acid-acetone extraction (Frost et al, 1996). Intra- and inter-assay coefficients of variations in our lab were 3.5% and 6.2% for IGF-I and 5.5% and 12.9% for IGF-II, respectively. Free IGF-I was measured by an immunoradiometric assay kit purchased from Diagnostic System Laboratories (Webster, TX) according to the manufacturers instructions. Intra-assay coefficient of variation in our control plasma pool from postmenopausal women was 13%.

IGFBP-3 proteolysis was measured indirectly by Western immunoblots as IGFBP-3 fragmentation (Frost et al, 1996). This was defined as the ratio of the major IGFBP-3 fragment (30 kDa) to total IGFBP-3 evaluated by densitometric scanning of immunoblots for IGFBP-3. The smaller occasional IGFBP-3 fragments of 20 and 16 kDa were not taken into account when calculating IGFBP-3 proteolysis. Intra- and inter-assay coefficients of variations in our control plasma pool were 4.6% and 15.5% respectively.

### Statistics

Values of the different IGF-parameters measured in normal pre- and postmenopausal women were tested separately for their distribution with use of Quartile-Quartile (Q-Q) plots (Johnson and Wichern, 1982). All parameters were found to be best fitted to a log normal distribution with the exception of the IGFBP-3 proteolysis, which was best described by a normal distribution. Thus, parameters obtained in the different groups of patients are given as their geometric mean value with 95% confidence intervals of the mean, with the exception of IGFBP-3 proteolysis where the arithmetic mean values are given. Thus, the normal range (95% confidence interval) of the individual observations for the plasma IGFBP-3 proteolysis was defined as a ratio of fragmented to total IGFBP-3 of 0.26–0.52 for premenopausal women and 0.22–0.58 for postmenopausal women. Patients having values above the normal range (0.52 for premenopausal and 0.58 for postmenopausal women) were considered to have an elevated plasma IGFBP-3 proteolysis. The Chi-square test including Yates correction for small numbers was used to compare the frequency of elevated IGFBP-3 fragmentation among patients with invasive breast carcinomas (stage I–IV). IGF values obtained from patients with increased IGFBP-3 fragmentation and those obtained from patients with normal protease activity were compared with use of the Mann-Whitney Rank-Sum Test. Correlations between different parameters were tested for using the SYSTAT program on a Macintosh computer. Univariate analyses were done using the Pearson correlation coefficient, while multivariate regression was performed using a general linear model following appropriate testing for co-linearity based on eigenvalues.

*© 2001 Cancer Research Campaign*

**Table 2** Pretreatment values of IGF parameters according to stage. Values are given (with the exception of IGFBP-3 proteolysis) as geometric mean with 95% confidence intervals

| | Benign/DCIS | Stage I | Stage II | Stage III | Stage IV |
|---|---|---|---|---|---|
| IGF-I (nmol $l^{-1}$) | 14.7 (11.2–19.5) | 10.7 (8.3–13.9) | 11.2 (9.9–12.6) | 13.6 (11.9–15.6) | 11.0 (7.7–15.9) |
| free IGF-I (nmol $l^{-1}$) | 0.42 (0.31–0.56) | 0.36 (0.28–0.47) | 0.29 (0.23–0.35) | 0.39 (0.31–0.48) | 0.35 (0.23–0.52) |
| Ratio free IGF-I / total IGF-I (%) | 2.8 (2.2–3.7) | 2.7 (2.1–3.4) | 2.5 (2.1–3.1) | 1.7 (1.4–2.2) | 1.7 (0.9–3.1) |
| IGF-II (nmol $l^{-1}$) | 67.8 (56.4–86.5) | 65.5 (54.9–78.1) | 61.2 (56.8–65.9) | 66.0 (59.4–73.2) | 64.6 (51.3–81.3) |
| IGFBP-3 proteolysis[a] | 0.38 (0.33–0.42) | 0.36 (0.33–0.39) | 0.42 (0.38–0.46) | 0.47 (0.41–0.53) | 0.63 (0.50–0.77) |

[a]Given as the ratio of fragmented to total IGFBP-3.

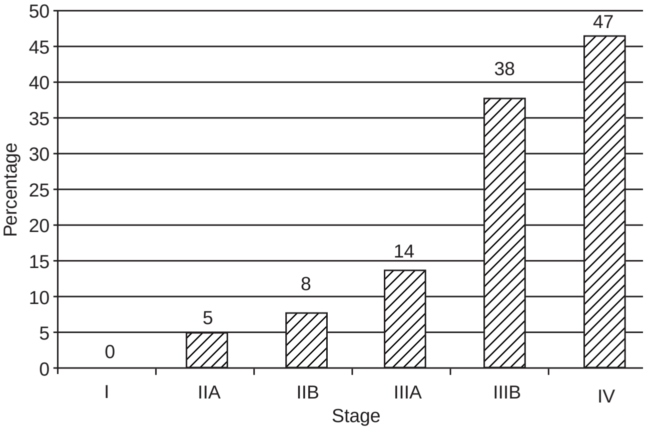

**Figure 1** The percentage of patients according to stage with elevated IGFBP-3 proteolysis (above the 95% confidence interval of the individual observations in our normal population of healthy premenopausal and postmenopausal women)

### RESULTS

### IGFBP-3 proteolysis in stage I–IV breast cancer and patients with DCIS/benign breast pathology

Mean values of different IGFP parameters according to stage are given in Table 2. 19 out of 116 (16%) patients suffering from breast cancer but 0 of 12 patients with DCIS/benign conditions expressed elevated IGFBP-3 proteolysis. Increased IGFBP-3 proteolysis was recorded among 0/19 (0%) of patients in Stage I, 3/45 (7%) in Stage II, 9/37 (24%) in stage III, and 7/15 (47%) in stage IV. The difference between all the stages were statistically significant ($P < 0.001$) also when comparing stage I, II and III ($P = 0.01$). The fraction of patients with increased IGFBP-3 proteolysis increased with increasing tumour stage (Figure 1). Patients expressing increased IGFBP-3 proteolysis had lower levels of IGF-I ($P < 0.001$), IGF-II ($P < 0.001$) but no difference were observed regarding free IGF-I compared to those with 'normal' IGFBP-3 proteolysis. Excluding the 7 patients suffering from conditions which possibly could influence IGFBP-3 proteolysis (rheumatoid arthritis: $n = 2$, type II diabetes; $n = 4$, or ulcerative colitis; $n = 1$) had no influence on the statistical results.

### Correlations between different IGF-parameters and demographic variables before treatment: univariate analysis

Table 3 shows univariate correlations between selected IGF-parameters and demographic variables in all patients. A strong positive correlation ($P < 0.001$) was observed between tumour stage and patient age on the one side and IGFBP-3 proteolysis. Total IGF-I levels correlated negatively both to age and IGFBP-3 proteolysis ($P < 0.001$), while the ratio of free to total IGF-I correlated positively ($P < 0.01$) to the same parameters. A significant correlation ($P < 0.001$) was also observed between the levels of total and free plasma IGF-I. IGF-II correlated negatively to IGFBP-3 proteolysis ($P < 0.001$) and age ($P < 0.05$), but neither IGF-II nor free IGF-I correlated to tumour stage.

### Multivariate analysis

IGFBP-3 proteolysis was positively correlated to age and tumour stage in multivariate analysis ($P < 0.01$), while total IGF-I levels correlated negatively to age and IGFBP-3 proteolysis ($P < 0.01$ for both). IGF-II correlated negatively to IGFBP-3 proteolysis ($P < 0.01$ for both). No significant correlation between free IGF-I and any of the parameters was seen. Demographic and tumour-related parameters like weight, body mass index or oestrogen receptor status did not correlate to any of the IGF-parameters.

### DISCUSSION

In previous studies we found IGFBP-3 fragmentation to be increased in a significant number of patients with metastatic breast

**Table 3** Univariate correlations (Pearson R-values) between IGF-parameters and tumour and demographic characteristics of interest

| | IGFBP-3 proteolysis | IGF-I | IGF-II | free IGF-I /total IGF-I |
|---|---|---|---|---|
| Stage | 0.408[a] | 0.022 | 0.016 | –0.13 |
| Age | 0.354[a] | –0.367[a] | –0.181[c] | 0.283[b] |
| Tumour grade | 0.148 | –0.015 | 0.028 | 0.055 |
| Oestrogen receptor status | –0.115 | –0.033 | 0.023 | –0.004 |
| Weight | –0.056 | 0.107 | 0.094 | –0.086 |
| IGFBP-3 proteolysis | – | –0.357[a] | –0.349[a] | 0.303[a] |

[a]$P < 0.001$; [b] $P < 0.01$; [c]$P < 0.05$.

*© 2001 Cancer Research Campaign*

cancer (Frost et al, 1996) and also to increase or decrease in relation to changes in tumour burden in these patients expressing elevated protease activity (Frost et al, 1996; Helle et al, 1996). The major aim of this study was to evaluate whether IGFBP-3 proteolysis also may be elevated in patients suffering from primary breast cancers with no evidence of metastasis and, if so, to evaluate a potential correlation between tumour burden and IGFBP-3 proteolysis. In addition, we wanted to evaluate the effect of elevated IGFBP-3 proteolysis on free IGF-I levels, which has not been determined in cancer patients previously.

In this study, we found elevated plasma IGFBP-3 proteolysis in 12 out of 101 patients (12%) with primary breast cancer without detectable metastases. The finding of an increasing number of patients with elevated IGFBP-3 proteolysis among patients with larger tumours supports our hypothesis that tumour burden may influence IGFBP-3 proteolysis, and that this relationship also holds in patients without overt metastatic disease. 47% of the patients with metastatic disease at time of diagnoses had elevated IGFBP-3 proteolysis. In a previous study 73% of patients with metastatic breast cancer had increased IGFBP-3 protease activity in a protease assay (using recombinant IGFBP-3) compared to normal sera (Frost et al, 1996). However, many of the patients in that study had a larger tumour burden and receiving second or third line therapy, which may explain a larger number with increased IGFBP-3 proteolysis.

The impact of alterations in IGFBP-3 proteolysis on the disposition of IGF-I and IGF-II is difficult to interpret. We observed a decrease in both IGF-I and -II but no difference in free IGF-I levels. Based on these data, it is difficult to assess the effect of elevated IGFBP-3 proteolysis on IGF-bioavailability. However, if an elevated IGFBP-3 proteolysis reflects an increased enzyme activity in the tissue it is tempting to speculate that the net effect is an elevated delivery of IGF-I to the tumour cells. If IGFBP-3 proteolysis acts as a growth accelerator, this could potentially explain why some tumours achieve a stage of accelerated growth during the process and also why a heavy tumour burden has been associated with a poor response to therapy (Swenerton et al, 1979). Further studies are warranted evaluating the prognostic (and possibly predictive) value of IGFBP-3 proteolysis in breast cancer patients.

Other investigators have previously reported increased total IGF-I levels and an increased IGF-I/IGFBP-3 ratio in premenopausal women with breast cancer (Bruning et al, 1995). High total IGF-I level has recently been found to be associated with an increased risk for development of breast cancer in premenopausal women (Hankinson et al, 1998). While total IGF-I may be a valid parameter for bioavailable IGF-I in healthy individuals, our study indicates that analysis of total IGF-I levels should be interpreted with caution in patients with manifest breast cancer or other serious illnesses causing increased IGFBP-3 proteolysis.

We conclude that a certain number of patients with primary breast cancer express elevated protease activity for IGFBP-3, and that this elevation correlates to tumour burden. The patient group with increased IGFBP-3 proteolysis has lower levels of IGF-I and -II but no difference in free IGF-I levels compared to the group with normal IGFBP-3 proteolysis.

### ACKNOWLEDGEMENTS

This work was supported by grants from the Norwegian Cancer Society. The technical assistance of Mr Dagfinn Elese is highly appreciated.

### REFERENCES

Bereket A, Lang CH, Blethen SL, Fan J, Frost RA and Wilson TA (1995) Insulin-like growth factor binding protein-3 proteolysis in children with insulin-dependent diabetes mellitus: a possible role for insulin in the regulation of IGFBP-3 protease activity. *J Clin Endocrinol Metab* **80**: 2282–2288

Blat C, Villaudy J and Binoux M (1994) In vivo proteolysis of serum insulin-like growth factor (IGF) binding protein-3 results in increased availability of IGF to target cells. *J Clin Invest* **93**: 2286–2290

Bruning PF, van Doorn J, Bonfrer JMG, van Noord PAH, Korse CM, Linders TC and Hart AAM (1995) Insulin-like growth factor binding protein 3 is decreased in early-stage operable pre-menopausal breast cancer. *Int J Cancer* **62**: 266–270

Chan JM, Stampfer MJ, Giovannucci E, Gann PH, Ma J, Wilkinson P, Hennekens CH and Pollak M (1998) Plasma insulin-like growth factor-I and prostate cancer risk: a prospective study. *Science* **279**: 563–566

Cotterill AM, Mendel P, Holly JMP, Timmins AG, Camacho-Hubner C, Cwyfan-Hughes S, Ross RMJ, Blum WF and Langford RM (1996) The differential regulation of the circulating levels of the insulin-like growth factors and their binding proteins (IGFBP) 1,2 and 3 after elective abdominal surgery. *Clin Endocrinol* **44**: 91–101

Davies SC, Wass JAH, Ross RJM, Cotterill AM, Buchanan CR, Coulson VJ and Holly JMP (1991) The induction of a specific protease for insulin-like growth factor binding protein-3 in the circulation during severe illness. *J Endocrinol* **130**: 469–473

Frost VJ, Helle SI, Lønning PE, van der Stappen JWJ and Holly JMP (1996) Effects of treatment with megestrol acetate, aminoglutethimide or formestane on insulin-like growth factor (IGF) I and II, IGF-binding proteins (IGFBPs) and IGFBP-3 protease status in patients with advanced breast cancer. *J Clin Endocrinol Metab* **81**: 2216–2221

Guler H-P, Zapf J, Schmid C and Froesch ER (1989) Insulin-like growth factors I and II in healthy man. Estimation of half-lives and production rates. *Acta Endocrinol* **121**: 753–758

Hankinson SE, Willett WE, Colditz GA, Hunter DJ, Michaud DS, Deroo B, Rosner B, Speizer FE and Pollak M (1998) Circulating concentrations of insulin-like growth factor-I and risk of breast cancer. *Lancet* **351**: 1393–1396

Helle SI, Holly JMP, Tally M, Hall K, van der Stappen J and Lønning PE (1996) Influence of treatment with tamoxifen and change in tumour burden on the IGF-system in breast cancer patients. *Int J Cancer* **69**: 335–339

Huynh H, Nickerson T, Pollak M and Yang X (1996) Regulation of insulin-like growth factor I receptor expression by the pure antiestrogen ICI 182780. *Clin Cancer Res* **2**: 2037–2042

Johnson RA and Wichern DW (1982) *Applied multivariate statistical analysis*. Prentice-Hall: Englewood Cliffs, New Jersey

Jones JI and Clemmons DR (1995) Insulin-like growth factors and their binding proteins: biological actions. *Endocrine Rev* **16**: 3–34

Karey KP and Sirbasku DA (1988) Differential responsiveness of human breast cancer cell lines MCF-7 and T47D to growth factors and 17β-estradiol. *Cancer Res* **48**: 4083–4092

Peyrat J-P, Bonneterre J, Beuscart R, Djiane J and Demaille A (1988) Insulin-like growth factor receptors in human breast cancer and their relation to estradiol and progesterone receptors. *Cancer Res* **48**: 6429–6433

Peyrat JP, Bonneterre J, Hecquet B, Vennin P, Louchez MM, Fournier C, Lefebvre L and Demaille A (1993) Plasma Insulin-like growth factor-I (IGF-I) concentrations in human breast cancer. *Eur J Cancer* **29A**: 492–497

Resnicoff M, Burgaud J-L, Rotman HL, Abraham D and Baserga R (1995) Correlation between apoptosis, tumorigenesis, and levels of insulin-like growth factor I receptors. *Cancer Res* **55**: 3739–3741

Swenerton KD, Legha SS, Smith T, Hortobagyi GN, Gehan EA, Yap H-Y, Gutterman JU and Blumenschein GR (1979) Prognostic factors in metastatic breast cancer treated with combination chemotherapy. *Cancer Res* **39**: 1552–1562

Turner BC, Haffty BG, Narayanan L, Yuan J, Havre PA, Gumbs AA, Kaplan L, Burgaud J-L, Carter D, Baserga R and Glazer PM (1997) Insulin-like growth factor-I receptor overexpression mediates cellular radioresistance and local breast cancer recurrence after lumpectomy and radiation. *Cancer Res* **57**: 3079–3083

Wiseman LR, Johnson MD, Wakeling AE, Lykkesfeldt AE, May FEB and Westley BR (1993) Type I IGF receptor and aquired tamoxifen resistance in oestrogen-responsive human breast cancer cells. *Eur J Cancer* **29A**: 2256–2264

© 2001 Cancer Research Campaign 
